# Dietary tributyrin attenuates intestinal damage induced by dextran sulfate sodium and modulates growth performance in broilers

**DOI:** 10.1016/j.psj.2026.107471

**Published:** 2026-07-18

**Authors:** Gabriel R. Braga, Maria R.S. Farias, Lucimauro Fonseca, Mariana A. Nascimento, Wender V. Fernandes, Ana L.C. Barros, Fernanda S. Marks, Lukas Bauer, Victor D. Naranjo, Melissa I. Hannas

**Affiliations:** a Department of Animal Science, Federal University of Viçosa, Viçosa-MG, Brazil; b Department of Veterinary Medicine, Federal University of Viçosa, Viçosa-MG, Brazil; c Evonik Operations GmbH, Hanau Wolfgang, Germany; d Evonik Guatemala, Guatemala City 01010, Guatemala

**Keywords:** Butyrate, Additive, Enteritis, Chicken, DSS

## Abstract

Enteric damage compromises intestinal integrity and may impair growth performance in broiler chickens. Dietary tributyrin has emerged as a promising nutritional strategy to preserve gut health. The present study evaluated the effects of tributyrin supplementation in mitigating DSS-induced intestinal damage in broilers. A total of 1200 male broilers were distributed in a completely randomized design with 5 treatments, 12 replicates, and 20 birds each. The treatments were: no challenge and basal diet (**PC**); challenge and basal diet (**NC**); NC + 1000 g/t of tributyrin A (**TBA0.1**); NC + 500 g/t of tributyrin A (**TBA0.05**) and NC + 500 g/t of tributyrin B (**TBB0.05**). Intestinal damage was induced by oral gavage of a 3.5% DSS solution between 8 and 12 and 21-25 days of age of the birds. Body weight (**BW**), body weight gain (**BWG**), average feed intake (**AFI**) and feed conversion ratio (**FCR**) were measured at d 8, 13, 21, 26 and 40. Samples from the duodenum, jejunum, ileum, and cecum were collected on days 13, 21 and 26 for histological and morphometric analyses, and for determination of digesta pH. Cecal contents were collected to measure short-chain fatty acid (**SCFA**) concentrations at d 26 and 40. Variables with a normal distribution were subjected to analysis of variance **(ANOVA**) followed by the Student-Newman-Keuls **(SNK)** test, while non-normal variables were analyzed using the Kruskal-Wallis and Dunn tests (P < 0.05). Treatments with tributyrin A showed better results for growth performance (P < 0.05) from 1 to 8 days and from 1 to 26 days, in addition to an increase in enterocyte proliferation and epithelial thickness (P < 0.05). Treatment with TBA0.1 promoted better responses in intestinal morphometry (P < 0.05) in all periods evaluated. The greatest pH differences were observed (P < 0.05) in the ileum. The concentration of SCFA was not affected (P > 0.05). Supplementation with 0.1% tributyrin proved to be an effective nutritional strategy for improving intestinal morphology and productive performance in broiler chickens.

## Introduction

The growth performance of modern broiler breeds has improved remarkably in recent years, as evidenced by faster growth rates and superior feed efficiency (Hou et al., 2023). This progress depends on adequate intestinal function, as the gastrointestinal tract is the primary site of nutrient digestion and absorption, enabling birds to fully express their genetic potential (Gao et al., 2025; Amer et al., 2021; Gazoni et al., 2020). However, the high metabolic demands associated with rapid growth increase the physiological burden on the gastrointestinal tract, making the intestinal mucosa more susceptible to damage caused by different stressors. Consequently, strategies aimed at preserving intestinal morphology and function have become a major focus of poultry research (Hu et al., 2021).

Experimental models of intestinal injury are widely used to investigate mechanisms of intestinal damage under controlled conditions. Among these models, dextran sulfate sodium (**DSS**) is a sulfated polysaccharide that induces epithelial injury, disrupts the mucosal barrier, and facilitates the translocation of luminal bacteria and antigens into intestinal tissues (Zou et al., 2018; Liu et al., 2022). DSS has been widely used to induce intestinal injury in several species, including pigs, mice, and broilers (Liu et al., 2022; Tajasuwan et al., 2023; Li et al., 2025; Xie et al., 2025). Moreover, the severity and duration of intestinal injury can be modulated by adjusting the dose and administration period (Yang et al., 2024).

In this context, butyrate, a short-chain fatty acid (**SCFA**), has received considerable attention because it serves as the primary energy substrate for intestinal epithelial cells and contributes to the maintenance of intestinal structure, regulation of epithelial cell proliferation, and inflammatory homeostasis (Melaku et al., 2021; Chen et al., 2024; Salvi et al., 2021; Sotira et al., 2020; Elnesr et al., 2018; Moquet et al., 2018; Silva et al., 2018). However, due to its volatility, unpleasant odor, and rapid absorption in the upper gastrointestinal tract, free butyrate supplementation alone may limit its biological efficacy (Ficagna et al., 2022; Gong et al., 2021; Xiong et al., 2018).

Tributyrin, a butyrate derivative, is more resistant to gastrointestinal conditions and releases butyrate throughout the intestinal tract in a controlled manner, preventing its immediate absorption in the upper gastrointestinal tract and, therefore, allowing more butyrate to reach the distal small intestine (Hu et al., 2022, 2021; Wang et al., 2021).

Despite the beneficial effects of tributyrin supplementation, studies evaluating its efficacy in large scale experimental models of intestinal damage induced by dextran sulfate sodium are still scarce. Furthermore, it remains unclear how tributyrin-mediated effects on enteric damage influence intestinal integrity across different segments of the gastrointestinal tract. Given this, we hypothesized that dietary tributyrin supplementation would attenuate DSS-induced enteric damage, thereby preserving intestinal morphology and sustaining broiler growth performance. The aim of this study was to evaluate the effect of dietary tributyrin supplementation on intestinal damage and growth performance in broilers challenged with oral administration of dextran sulfate sodium.

## Materials and methods

All procedures involving animal care and use were approved by the Institutional Animal Use Committee of the Federal University of Viçosa, Viçosa, Minas Gerais, Brazil (**CEUAP**-031/2024), and followed the standards outlined by the National Council for the Control of Animal Experimentation (**CONCEA**).

### Experimental design, diets and animals

One thousand two hundred 1-d-old male Cobb 500® broiler chickens (44.4 ± 0.1 g) were distributed in a completely randomized design with five treatments, twelve replicates, and twenty birds per experimental unit. The birds were housed in a masonry shed with side curtains, in a concrete box measuring 0.6 m × 1.5 m × 2.0 m (height × width × length), with a wood shavings floor. Each box was considered an experimental unit. A photoperiod of 12 hours of light was adopted until the 14th day, and after that period a photoperiod of 18 hours of light: 6 hours of darkness was adopted. The house temperature was recorded five times a day using thermometers placed at the height of the birds' body mass and adjusted as needed by operating curtains and fans. The experimental period consisted of four phases: the first from 1 to 8 days, the second from 9 to 21 days, the third from 22 to 35 days, and the fourth from 36 to 40 days. The birds received feed and water ad libitum throughout the experimental period, from 1 to 40 days of age.

### Experimental treatments

The experimental diets were formulated based on corn and soybean meal, supplemented with minerals and vitamins according to the recommendations of Hannas et al. (2024) (Table 2). The experiment was conducted with five distinct treatments, involving different levels of tributyrin supplementation. Two commercial tributyrin products were used, namely tributyrin A (**TBA**; Evonik, Germany), which contains butyric acid in an esterified form as tributyrin (60%) on a silicon dioxide carrier (35%), and tributyrin B (**TBB**; Perstorp, Sweden), which contains butyric acid in an esterified form mainly as diglycerides and triglycerides (62%) on a silicon dioxide carrier (32%). Both products were included in the diets according to the levels established for each treatment.

The dietary treatments (Table 1) consisted of: positive control **(PC)** – no challenge and basal diet; negative control **(NC)** – with challenge and basal diet; **TBA0.1** – with challenge and supplementation of 1000 g/t of tributyrin A; **TBA0.05** – with challenge and supplementation of 500 g/t of tributyrin A; and **TBB0.05** – with challenge and supplementation of 500 g/t of tributyrin B.

**Table 1:** Description of experimental treatments applied in the experiment.

Treatment	Abbreviation	Challenge	Diet
Positive Control	PC	No challenge with DSS	Basal diet (Hannas et al., 2024)
Negative Control	NC	3.5% DSS by oral gavage on experimental days 8-12 and 21-25	Basal diet (Hannas et al., 2024)
Tributyrin A	TBA0.1	3.5% DSS by oral gavage on experimental days 8-12 and 21-25	Basal diet (Hannas et al., 2024), with supplementation of 0,1% of tributyrin A
Tributyrin A	TBA0.05	3.5% DSS by oral gavage on experimental days 8-12 and 21-25	Basal diet (Hannas et al., 2024), with supplementation of 0,05% of tributyrin A
Tributyrin B	TBB0.05	3.5% DSS by oral gavage on experimental days 8-12 and 21-25	Basal diet (Hannas et al., 2024), with supplementation of 0,05% da tributyrin B

DSS = dextran sulfate sodium; TBA = tributyrin A and TBB = tributyrin B.

**Table 2:** Ingredient and calculated nutrient composition of experimental diets for broiler chickens.

Ingredients (%)		Experimental Phases (days)			
		1-8	8-21	21-35	35-40
Corn 7,88%		449.60	476.51	539.62	567.17
Soybean Meal 46%		466.68	441.83	388.98	360.84
Soy Oil		33.00	35.05	36.35	38.80
Dicalcium Phosphate		23.40	20.50	12.20	10.60
Calcitic Limestone		11.20	10.20	7.30	6.90
Salt		5.25	5.05	4.85	4.60
Starch		1.50	1.50	1.50	1.50
Vitamin Premix^1^		1.45	1.36	1.00	0.90
Micromineral Premix^2^		1.45	1.36	1.00	0.90
Antioxidant^3^		0.50	0.50	0.50	0.50
Lysine Sulphate, 60%		1.25	1.40	2.10	2.55
DL-Methionine, 99%		3.28	3.37	3.39	3.43
L – Threonine, 99%		0.40	0.36	0.44	0.52
L – Valine, 99%		-	-	0.03	0.18
Choline Chloride, 60%		1.04	1.01	0.74	0.61
**Total**		**1000.00**	**1000.00**	**1000.00**	**1000.00**
**Nutritional levels**^4^					
Met. Energy	Kcal/kg	2.950,00	3.000,00	3.100,00	3.150,00
Fat	%	6.33	6.56	6.78	7.05
Crude Protein	%	25.54	24.61	22.73	21.68
Digestible Lysine	%	1.37	1.31	1.23	1.18
Digestible Methionine	%	0.66	0.66	0.64	0.63
Methionine + Digestible Cystine	%	1.00	0.99	0.95	0.92
Digestible Threonine	%	0.90	0.87	0.81	0.78
Digestible Tryptophan	%	0.30	0.29	0.26	0.25
Digestible Arginine	%	1.64	1.57	1.42	1.34
Digestible Glycine + Serine	%	2.01	1.93	1.77	1.68
Digestible Valine	%	1.07	1.03	0.95	0.91
Digestible Isoleucine	%	1.01	0.97	0.88	0.83
Digestible Leucine	%	1.90	1.85	1.74	1.67
Digestible Histidine	%	0.61	0.59	0.54	0.52
Digestible Phenylalanine	%	1.16	1.11	1.02	0.97
Phenylalanine + Tyrosine	%	2.00	1.93	1.77	1.68
Total Calcium	%	1.17	1.05	0.72	0.66
Total Phosphorus	%	0.82	0.75	0.58	0.55
Available Phosphorus	%	0.56	0.50	0.34	0.31
Sodium	%	0.22	0.21	0.21	0.20

1 Composition per kg of product: vit. A, 10.824.000 UI; vit. D3, 2.651.000 UI; vit. E, 40.600 UI; vit. B1, 2.690 mg; vit B2, 6.700 mg; vit. B6, 3.760 mg; vit B12, 20,1 mg; vit K3, 2.29 mg; pantothenic acid, 14.11 mg; Folic acid, 1.09 mg; niacin acid, 44.80 mg; biotin, 104,1 mg.

2 Composition per kg of product: copper, 9.48 g; iron, 45.41 g; iodine 0,96 g; manganese, 66,47 g; selenium, 0,28 g and zinc, 62.32 g.

3 Hydroxybutyltoluene – BHT;.

4 Calculated values were based on the ingredient's nutritional composition (Rostagno et al., 2024).

### Intestinal challenge

Intestinal challenge was induced by oral gavage of dextran sulfate sodium (Sigma-Aldrich, St. Louis, MO, USA) with a molecular weight of 50 kDa, at the same time for five consecutive days in the second phase (8 to 12 days) and third phase (21 to 25 days) of the experiment. All birds in the experimental units designated for the intestinal challenge received a 3.5% DSS solution, and the birds in the PC treatment were also subjected to the oral gavage procedure, on the same days and times, in order to standardize the experimental management; however, in this group, only distilled water was administered.

The DSS concentration and challenge periods were selected based on previously published studies (Liu et al., 2022; Dal Pont et al., 2021; Menconi et al., 2015), which validated this protocol as an experimental model capable of inducing moderate, consistent, and reproducible intestinal lesions in broiler chickens. This DSS concentration induces controlled histopathological and morphological alterations in the intestinal mucosa, making it a suitable model for evaluating the efficacy of nutritional strategies aimed at preserving intestinal integrity. The challenges were conducted from 8 to 12 and from 21 to 25 days of age to induce intestinal injury during two distinct stages of broiler development, allowing the evaluation of tributyrin effects during both the acute response to the challenge and the subsequent periods of intestinal mucosal recovery. It should be noted that the experimental model adopted consisted of the chemical induction of intestinal challenge, not involving the use of pathogens, infectious agents or microbiological challenges, and is therefore a controlled and reproducible model used to simulate inflammatory changes in the intestinal tract.

### Growth performance

All experimental diets and birds were weighed at 8, 13, 21, 26, and 40 days of age to calculate body weight (**BW, g**), body weight gain (**BWG, g/bird**), the average feed intake (**AFI, g/bird**) and feed conversion ratio (**FCR, g/g**). Mortalities were recorded each day of the experiment for subsequent intake corrections.

### Sample collection

Samples were collected at 13, 21, 26, and 40 days of age from one bird per experimental unit (12 birds per treatment), selected based on the average weight of the group. Birds were euthanized by cervical dislocation following Brazilian regulations on animal experimentation, and samples were collected after euthanasia. Samples of the duodenum, jejunum, ileum, and cecum were collected for histological analyses. Intestinal anatomy was used as a reference to ensure sampling of similar segments in all birds. Duodenal samples (5 cm) were collected 1 cm caudal to the duodenal loop. Meckel's diverticulum was used to separate the jejunum and ileum, and sampling was performed in the middle of each segment (5 cm). One cecum was cleaned, and cecal digesta was collected for SCFA concentration analyses.

### Intestinal Histology

Jejunal, duodenal, ileal, and cecal sections were fixed in 10% neutral buffered formalin and embedded in paraffin, sectioned (5 mm thick), placed on a glass slide, and stained with hematoxylin and eosin **(H&E)** combined with alcian blue and periodic acid-Schiff **(PAS)**, and subsequently subjected to analysis by optical microscopy. Intestinal histology segment assessments were performed at 13, 21, and 26 days. For the evaluation of microscopic changes, 20 intestinal villi per slide were evaluated at 10X magnification (using 40X magnification to confirm the changes) using an optical microscope.

For the analysis of histological parameters, a numerical scoring system was adopted for the quantification of pathological changes, based on the I See Inside **(ISI)** methodology described in the literature (Belote et al., 2019). The ISI methodology consists of a numerical scoring system used to quantify changes observed in microscopic analysis. In this method, each histological alteration receives an impact factor **(IF)**, defined based on the possible reduction in the functional capacity of the evaluated tissue, considering information from scientific literature and experimental research. The IF can range from 1 to 3, with the value 3 assigned to alterations with the greatest functional impact. In addition, each lesion observed in the tissue is classified according to its intensity or extent, through a lesion score **(S)** that ranges from 0 to 3. A score of 0 indicates the absence of a lesion or the occurrence of the alteration; 1 corresponds to the presence of the alteration in up to 25% of the area or frequency observed; 2 represents alterations that cover from 25.1% to 50% of the area or frequency; and 3 indicates that the alteration is present in more than 50% of the area or frequency evaluated. The final ISI index is obtained by multiplying the IF by the S for each identified alteration.

The histological parameters evaluated included epithelial thickness, lamina propria thickness, enterocyte proliferation, and goblet cell proliferation.

### Intestinal morphometry

Morphometric observations of intestinal segment were performed at 13, 21, 26 and 40 days, using optical microscopy with the EVOS M5000 Imaging System (Thermo Fisher Scientific, Waltham, MA, USA) at 10× magnification. The images obtained were analyzed using ImageJ software (version 1.50i; National Institutes of Health, Bethesda, MD, USA).

For each bird, 20 intestinal villi and 20 crypts were measured, randomly selected from well-oriented histological sections. Villus height **(VH)** and crypt depth **(CD)** were determined, and the ratio between these variables **(VH:CD)** was subsequently calculated.

### Intestinal pH

During necropsies, digesta pH measurements were performed on samples collected from the duodenum, jejunum, ileum, and cecum. To achieve this, the gastrointestinal tract, containing the luminal contents, was carefully removed aseptically. Before emptying the contents, each segment of the gastrointestinal tract was clamped to avoid cross-contamination between segments. The pH of the luminal contents was measured using a digital pH meter (HI99161, Hanna Instruments). The fine end of the meter's glass electrode was sterilized before each measurement and carefully inserted into each segment, recording the corresponding pH.

### Short chain fatty acid concentration in the cecum

Acetate, propionate and butyrate concentrations were measured in cecal digesta samples collected at two different time points, at 26 and 40 days of age. Cecal content samples from one bird per experimental unit were collected, weighed and a phosphate buffer solution with a pH of 7.2 was added to the cecal content at a concentration of 1:9 (sample: solution). This mixture was then vortexed for 1 minute. The sample was then acidified with a 50% sulfuric acid solution and stored at −80°C for later analysis.

The analysis of SCFA concentration in the cecum was performed according to the methodology described by Siegfried et al. (1984), where, initially, the samples were thawed at room temperature. Then, an 800 μL aliquot of each sample was transferred to microtubes and centrifuged at 12,000 × g for 10 minutes. After this process, 600 μL of the supernatant were transferred to new microtubes, to which 600 μL of calcium hydroxide solution (Ca(OH)₂) and 300 μL of copper sulfate solution (CuSO₄) were added. The samples were homogenized in a vortex for 10 seconds and frozen.

After thawing at room temperature, a new centrifugation was performed at 12,000 × g for 10 minutes. Next, 1.0 mL of the supernatant was transferred to new microtubes, and then 28 μL of concentrated P.A. H₂SO₄ was added. The microtubes were subjected to successive freeze-thaw cycles (freeze, thaw, refreeze, and final thaw), followed by centrifugation at 12,000 × g for 10 minutes. At the end, 600 μL of the supernatant were transferred to HPLC vials and stored at 4°C until the time of analysis.

For the preparation of the analytical standards, the amounts of acids to be quantified were previously calculated. The corresponding masses were transferred to volumetric flasks, with the volume adjusted to 50 mL using ultrapure water. From these stock solutions, successive dilutions (1×, 2×, 4×, 8×, and 16×) were performed to construct the calibration curves. The standards were subjected to the same procedures described for the samples, ensuring standardization of preparation. Finally, they were transferred to HPLC vials and stored at 4°C until the time of analysis.

### Statistical analysis

Data were analyzed using a completely randomized design. For performance variables, each pen was considered the experimental unit, whereas for the other variables, each bird was considered the experimental unit. Residual normality was assessed using the Shapiro–Wilk test, and homogeneity of variances was assessed using Levene's test. Variables with a normal distribution were subjected to analysis of variance (ANOVA), and means were compared using the Student–Newman–Keuls (SNK) test. Continuous variables that did not meet normality assumptions, as well as variables expressed as ordinal scores, were analyzed using the Kruskal–Wallis test followed by Dunn's test for multiple comparisons.

Morphometric and histological analyses were performed separately for each age, since different birds were sampled and euthanized at each collection time point, constituting independent observations. Furthermore, each age represented a specific biological stage of the experimental protocol (acute response to the challenge or intestinal recovery period), rather than a repeated-measures design. All statistical analyses were performed using R software (version 4.4.0), adopting a significance level of 5%.

## Results

### Growth performance

The results of the zootechnical performance are presented in Table 3.

**Table 3:** Growth performance of broiler chickens challenged with dextran sulfate sodium and supplemented with tributyrin.

Phase	Parameters	Treatments					SEM^5^	p-value
		PC	NC	TBA0.1	TBA0.05	TBB0.05		
**1 d**	BW^1^	44.53	44.39	44.54	44.45	44.44	0.033	0.507
**1 – 8 d**	BW	228 ab	223 b	237 a	235 ab	234 ab	1.622	0.032
	BWG^2^	184	179	192	190	187	1.681	0.069
	AFI^3^	203	203	205	207	208	1.031	0.489
	FCR^4^	1.11	1.12	1.06	1.09	1.09	0.007	0.167
**1 – 13 d**	BW	479	479	490	489	488	2.910	0.550
	BWG	434	434	446	445	444	2.912	0.540
	AFI	504	508	503	515	502	2.417	0.427
	FCR	1.14 b	1.19 a	1.12 b	1.14 b	1.14 b	0.006	0.035
**1 – 21 d**	BW	1162	1154	1180	1191	1177	4.540	0.072
	BWG	1118	1110	1136	1147	1132	4.536	0.072
	AFI	1333	1323	1331	1382	1344	7.194	0.066
	FCR	1.19	1.20	1.17	1.20	1.18	0.003	0.093
**1 – 26 d**	BW	1722 ab	1686 b	1737 ab	1745 a	1730 ab	6.729	0.050
	BWG	1678 ab	1642 b	1692 ab	1700 a	1685 ab	6.718	0.050
	AFI	2088 ab	2026 b	2055 ab	2108 a	2067 ab	8.598	0.020
	FCR	1.23	1.23	1.22	1.23	1.21	0.041	0.593
**1 – 40 d**	BW	3535	3492	3507	3533	3520	9.321	0.578
	BWG	3491	3447	3463	3488	3475	9.316	0.578
	AFI	4880	4773	4837	4856	4792	15.689	0.167
	FCR	1.40	1.37	1.39	1.39	1.39	0.004	0.395

PC = basal diet without DSS challenge; NC = basal diet with 3.5% DSS challenge; TBA0. 1 = 3.5% DSS challenge + basal diet supplemented with 0.1% tributyrin A; TBA0. 05 = 3.5% DSS challenge + basal diet supplemented with 0.05% tributyrin A; TBB0. 05 = 3.5% DSS challenge + basal diet supplemented with 0.05% tributyrin B.

Data are presented as means of 12 replicate pens per treatment (one bird sampled per pen; n = 12 birds per treatment).

a,b Means within a row followed by different superscript letters differ according to SNK test (P < 0.05).

1 Body weight (g).

2 Body weight gain (g).

3 Average feed intake (g).

4 Feed conversion rate (g/g).

5 SEM = Standard error of the mean.

Birds in the TBA0.1 treatment showed higher body weight during the 1-to-8-day phase compared to birds in the NC treatment (P = 0.032). Birds in the NC treatment exhibited the worst feed conversion ratio during the 1-to-13-day phase compared to the other treatments (P = 0.035). Birds in the TBA0.05 treatment showed higher body weight and greater body weight gain (P = 0.050) and higher feed intake (P = 0.020) during the 1-to-26-day phase compared to birds in the NC treatment. During the 1-to-21-day and 1-to-40-day phases, performance variables were not influenced by the treatments (P > 0.05).

### Intestinal histology

The results of the histological parameters are presented below in Table 4.

**Table 4:** Histomorphological and histomorphometric parameters of the duodenum in broiler chickens challenged with dextran sulfate sodium and supplemented with tributyrin.

Age	Segment	Parameters^1^	Treatments						SEM^2^	p-value
			PC	NC	TBA0.1		TBA0.05	TBB0.05		
**13 d**	**Duodenum**	Lamina Proprium Thickness	1.92	1.90	1.87		1.90	1.95	0.051	0.657
		Epithelial Thickness	1.42	1.42	1.43		1.48	1.49	0.042	0.317
		Enterocyte Proliferation	1.02 b	1.09 ab	1.13 a		1.15 a	1.12 ab	0.022	0.006
		Goblet Cells	2.02	2.15	2.16		1.94	2.10	0.044	0.136
	**Jejunum**	Lamina Proprium Thickness	2.17 a	2.06 ab	1.62 c		1.96 b	1.91 b	0.070	<0.001
		Epithelial Thickness	1.35 a	1.28 a	1.10 b		1.12 b	1.17 b	0.043	<0.001
		Enterocyte Proliferation	1.13 a	1.08 a	0.95 b		1.05 ab	1.03 ab	0.031	<0.001
		Goblet Cells	2.10 a	2.16 a	1.79 b		1.67 b	1.83 b	0.052	<0.001
	**Ileum**	Lamina Proprium Thickness	2.48 b	2.41 b	2.55 ab		2.73 a	2.72 a	0.051	<0.001
		Epithelial Thickness	1.11 b	1.10 b	1.14 ab		1.20 a	1.21 a	0.031	<0.001
		Enterocyte Proliferation	0.95 a	0.82 c	0.87 abc		0.85 bc	0.94 ab	0.023	<0.001
		Goblet Cells	1.58	1.65	1.79		1.80	1.81	0.072	0.059
	**Cecum**	Enterocyte Proliferation	0.44 b	0.33 b	0.42 b		0.38 b	0.74 a	0.051	<0.001
**21 d**	**Duodenum**	Lamina Proprium Thickness	2.11	2.17	2.13	2.07		2.17	0.043	0.319
		Epithelial Thickness	1.50	1.47	1.45	1.45		1.47	0.045	0.826
		Enterocyte Proliferation	1.10 ab	1.07 b	1.18 ab	1.19 a		1.18 ab	0.022	0.003
		Goblet Cells	1.95 ab	1.95 ab	2.01 a	1.83 ab		1.76 b	0.052	0.02
	**Jejunum**	Lamina Proprium Thickness	2.04 ab	1.95 b	2.14 a	2.05 ab		2.07 ab	0.040	0.002
		Epithelial Thickness	1.19	1.18	1.27	1.20		1.24	0.030	0.122
		Enterocyte Proliferation	0.97 bc	0.92 c	1.11 a	1.04 ab		1.05 ab	0.034	<0.001
		Goblet Cells	1.91 ab	2.05 a	2.10 a	1.69 b		1.76 b	0.062	<0.001
	**Ileum**	Lamina Proprium Thickness	2.72	2.75	2.80	2.85		2.85	0.084	0.391
		Epithelial Thickness	1.12 c	1.10 c	1.22 ab	1.23 a		1.13 bc	0.024	<0.001
		Enterocyte Proliferation	0.86 ab	0.85 ab	0.88 ab	0.91 a		0.78 b	0.020	0.006
		Goblet Cells	1.41 bc	1.65 ab	1.73 a	1.46 abc		1.37 c	0.031	<0.001
	**Cecum**	Enterocyte Proliferation	0.42 c	0.49 bc	0.60 ab	0.63 a		0.69 a	0.040	<0.001
**26 d**	**Duodenum**	Lamina Proprium Thickness	2.05 ab	2.10 ab	2.15 a	2.09 ab		1.97 b	0.040	0.010
		Epithelial Thickness	1.40 ab	1.43 ab	1.42 ab	1.47 a		1.30 b	0.043	0.005
		Enterocyte Proliferation	1.20 a	1.13 ab	1.17 a	1.15 a		1.02 b	0.020	<0.001
		Goblet Cells	1.92	1.98	1.84	1.96		1.88	0.041	0.584
	**Jejunum**	Lamina Proprium Thickness	1.92	2.00	1.98	1.93		2.03	0.041	0.071
		Epithelial Thickness	1.29	1.23	1.29	1.30		1.25	0.040	0.485
		Enterocyte Proliferation	1.09	1.06	1.07	1.13		1.06	0.040	0.449
		Goblet Cells	2.39 a	2.10 b	2.24 ab	2.20 ab		2.13 ab	0.043	0.039
	**Ileum**	Lamina Proprium Thickness	2.74	2.84	2.69	2.91		2.86	0.061	0.076
		Epithelial Thickness	1.03 b	1.07 ab	1.11 ab	1.09 ab		1.12 a	0.023	0.024
		Enterocyte Proliferation	0.77	0.79	0.81	0.75		0.77	0.033	0.611
		Goblet Cells	1.34 c	1.65 ab	1.75 a	1.50 bc		1.55 abc	0.012	<0.001
	**Cecum**	Enterocyte Proliferation	0.63	0.69	0.62	0.61		0.60	0.052	0.267

PC = basal diet without DSS challenge; NC = basal diet with 3.5% DSS challenge; TBA0.1 = 3.5% DSS challenge + basal diet supplemented with 0.1% tributyrin A; TBA0.05 = 3.5% DSS challenge + basal diet supplemented with 0.05% tributyrin A; TBB0.05 = 3.5% DSS challenge + basal diet supplemented with 0.05% tributyrin B.

Data are presented as means of 12 replicate pens per treatment (one bird sampled per pen; n = 12 birds per treatment).

^a,b^ Means within a row followed by different superscript letters differ according to Dunn's test (P < 0.05).

1 ISI parameters were calculated using the I See Inside score methodology, based on the sum of the impact factor multiplied by the histopathological score [ISI = Σ(IF × S)]; therefore, values are expressed as dimensionless scores.

2 SEM = Standard error of the mean.

***Day 13.*** In the duodenum, birds supplemented with tributyrin A showed greater enterocyte proliferation than birds in the PC treatment (P = 0.006), whereas lamina propria thickness, epithelial thickness, and goblet cell number were not affected (P > 0.05). In the jejunum, TBA0.1 reduced lamina propria thickness (P < 0.001) and enterocyte proliferation (P < 0.001) compared with the PC treatment, while all tributyrin-supplemented groups showed lower epithelial thickness (P < 0.001) and goblet cell (P < 0.001) than the control treatments. In the ileum, supplementation with TBA0.05 and TBB0.05 increased lamina propria thickness (P < 0.001) and epithelial thickness (P < 0.001) compared with both control groups, whereas enterocyte proliferation was greater in the PC than in the NC treatment (P < 0.001), and globet cells was not affected (P > 0.05). In the cecum, birds receiving TBB0.05 showed the highest enterocyte proliferation among treatments (P < 0.001).

***Day 21.*** In the duodenum, birds receiving TBA0.05 showed greater enterocyte proliferation (P = 0.003) than those in the NC treatment, and TBA0.1 increased goblet cell number (P = 0.02) compared with TBB0.05, whereas lamina propria thickness, and epithelial thickness were not affected (P > 0.05). In the jejunum, TBA0.1 increased lamina propria thickness (P = 0.002) and enterocyte proliferation (P < 0.001) relative to NC birds, while TBA0.05 and TBB0.05 reduced goblet cell numbers (P < 0.001) compared with NC and TBA0.1, whereas epithelial thickness was not affected (P > 0.05). In the ileum, TBA0.05 increased epithelial thickness (P < 0.001) compared with both control treatments, TBA0.1 increased goblet cell number (P < 0.001) compared with TBB0.05, and enterocyte proliferation was greater in TBA0.05 than in TBB0.05 (P = 0.006), whereas lamina propria thickness was not affected (P > 0.05). In the cecum, enterocyte proliferation was higher in birds supplemented with TBA0.05 and TBB0.05 (P < 0.001) than in birds from the PC treatment.

***Day 26.*** In the duodenum, birds receiving TBA0.1 and TBA0.05 showed greater lamina propria thickness (P = 0.010) and epithelial thickness (P = 0.005) than those in the TBB0.05 treatment respectively, and TBB0.05 and TBB0.05 reduced the enterocyte proliferation (P < 0.001) compared to the other treatments, whereas globet cells were not affected (P > 0.05). In the jejunum, only goblet cell number differed among treatments, with birds from the PC treatment showing higher values (P = 0.039) than birds from the NC treatment. In the ileum, TBB0.05 increased epithelial thickness (P < 0.024) relative to the PC treatment, and birds receiving TBA0.1 showed a greater number of goblet cells (P < 0.001) than those receiving the PC treatment, whereas lamina proprium thickness and enterocyte proliferation were not affected (P > 0.05). No significant differences in enterocyte proliferation were detected in the cecum (P > 0.05).

### Intestinal morphometry

The results of the morphometric parameters are presented below, in Table 5.

**Table 5:** Intestinal morphometric parameters of broiler chickens challenged with dextran sulfate sodium and supplemented with tributyrin.

Age	Segment	Variable	Treatments					SEM^1^	p-value
			PC	NC	TBA0.1	TBA0.05	TBB0.05		
**/13 d**	**Duodenum**	VH	1288 b	1184 c	1328 a	1291 b	1265 b	7.881	<0.001
		CD	166 cd	162 d	174 bc	177 b	186 a	1.733	<0.001
		VH:CP	7.65 a	7.36 a	7.46 a	7.31 a	6.81 b	0.072	<0.010
	**Jejunum**	VH	642 ab	548 c	666 a	613 b	612 b	7.565	<0.001
		CD	127 a	100 b	125 a	131 a	135 a	2.284	<0.001
		VH:CP	5.03 ab	5.46 a	5.31 a	4.74 b	4.27 c	0.081	<0.001
	**Ileum**	VH	502 a	431 b	454 b	438 b	430 b	7.875	0.010
		CD	138 a	103 c	134 a	118 b	116 b	2.616	<0.001
		VH:CP	3.70 a	4.02 a	3.29 b	3.82 a	3.82 a	0.060	<0.001
	**Cecum**	VH	239 a	210 c	236 a	227 ab	219 bc	2.129	<0.001
		CD	102 c	124 a	117 b	106 c	105 c	1.473	<0.001
		VH:CP	2.35 a	1.64 c	2.10 b	2.14 b	2.08 b	0.036	<0.001
**21 d**	**Duodenum**	VH	1350 a	1183 b	1365 a	1368 a	1360 a	13.709	<0.001
		CD	213 c	236 a	223 b	219 bc	236 a	1.665	<0.001
		VH:CP	6.56 a	5.03 d	6.01 bc	6.14 b	5.77 c	0.080	<0.001
	**Jejunum**	VH	864 a	669 c	819 a	769 b	758 b	11.525	<0.001
		CD	171 a	175 a	168 b	173 a	175 a	8.290	<0.001
		VH:CP	5.11 a	3.78 c	4.96 a	4.43 b	4.35 b	0.077	<0.001
	**Ileum**	VH	558 a	436 c	460 b	464 b	452 bc	6.737	<0.001
		CD	165 b	171 a	154 c	162 b	163 b	0.908	<0.001
		VH:CP	3.36 a	2.57 d	2.96 b	2.7 c	2.77 c	0.041	<0.001
	**Cecum**	VH	285 a	189 d	233 b	223 bc	217 c	4.550	<0.001
		CD	156 b	170 a	127 d	147 c	146 c	1.987	<0.001
		VH:CP	1.85 a	1.11 c	1.81 a	1.50 b	1.50 b	0.037	<0.001
**26 d**	**Duodenum**	VH	1655 b	1560 d	1704 a	1602 c	1597 c	17.203	<0.001
		CD	187 d	211 a	206 b	194 c	194 c	1.250	<0.001
		VH:CP	8.81 a	7.36 c	8.26 b	8.23 b	8.23 b	0.061	<0.001
	**Jejunum**	VH	958 a	750 c	971 a	872 b	848 b	11.557	<0.001
		CD	190 e	210 a	205 b	199 c	196 d	4.498	<0.001
		VH:CP	5.03 a	3.56 d	4.72 b	4.37 c	4.31 c	0.070	<0.001
	**Ileum**	VH	561 a	416 e	548 b	475 c	459 d	7.751	<0.001
		CD	140 e	150 a	145 b	144 c	143 d	0.461	<0.001
		VH:CP	3.98 a	2.76 e	3.76 b	3.31 c	3.20 d	0.061	<0.001
	**Cecum**	VH	251 a	188 e	231 b	215 c	212 d	2.849	<0.001
		CD	111 e	15 a	125 b	119 c	118 d	1.887	<0.001
		VH:CP	2.26 a	1.25 d	1.83 b	1.79 c	1.79 c	0.042	<0.001
**40 d**	**Duodenum**	VH	1449 ab	1319 c	1477 a	1369 bc	1422 ab	13.735	<0.001
		CD	197 b	193 b	206 ab	220 a	212 a	2.280	<0.001
		VH:CP	7.32 a	6.97 ab	6.94 ab	6.24 b	6.59 ab	0.100	0.005
	**Jejunum**	VH	1073	905	967	1034	1072	58.348	0.059
		CD	171 a	174 a	161 b	159 b	160 b	1.043	<0.001
		VH:CP	6.10 a	5.14 b	6.31 a	6.02 a	6.34 a	0.085	<0.001
	**Ileum**	VH	565	492	584	552	504	7.114	0.063
		CD	133	150	153	144	134	2.495	0.069
		VH:CP	4.12 a	3.07 c	3.51 b	3.89 ab	3.83 ab	0.073	<0.001
	**Cecum**	VH	452 a	374 b	429 a	287 c	289 c	9.205	<0.001
		CD	224 a	207 b	211 b	127 c	127 c	6.186	<0.001
		VH:CP	1.99 ab	1.76 b	1.95 ab	2.09 a	2.13 a	0.040	0.020

PC = basal diet without DSS challenge; NC = basal diet with 3.5% DSS challenge; TBA0.1 = 3.5% DSS challenge + basal diet supplemented with 0.1% tributyrin A; TBA0.05 = 3.5% DSS challenge + basal diet supplemented with 0.05% tributyrin A; TBB0.05 = 3.5% DSS challenge + basal diet supplemented with 0.05% tributyrin B.

Data are presented as means of 12 replicate pens per treatment (one bird sampled per pen; n = 12 birds per treatment).

VH= villus height (µm); CD = crypt depth (µm); VH:CD = ratio of villus height to crypt depth.

^ab^ Means within a row followed by different superscript letters differ according to Student–Newman–Keuls (SNK) test (P < 0.05).

1 Standard error of the mean.

***Day 13.*** TBA0.1 increased villus height in the duodenum (P < 0.001), whereas NC showed the lowest crypt depth (P < 0.001) and TBB0.05 the lowest VH:CD ratio (P < 0.010). In the jejunum, villus height was greater in the PC and TBA0.1 treatments (P < 0.001), while NC showed the lowest crypt depth (P < 0.001) and TBB0.05 showed the worse VH:CD ratio (P < 0.001). In the ileum, villus height was greatest in the PC treatment (P = 0.010), crypt depth was lowest in the NC treatment (P < 0.001), and TBA0.1 showed the lowest VH:CD ratio (P < 0.001). In the cecum, NC birds exhibited reduced villus height, greater crypt depth, and the lowest VH:CD ratio (P < 0.001).

***Day 21.*** Birds in the NC treatment showed the lowest villus height and the poorest VH:CD ratio in the duodenum (P < 0.001), whereas PC birds had the lowest crypt depth (P < 0.001). In the jejunum, villus height remained greater in the PC and TBA0.1 treatments (P < 0.001), while TBA0.1 showed the lowest crypt depth (P < 0.001) and NC the lowest VH:CD ratio (P < 0.001). In the ileum, birds in the PC treatment exhibited greater villus height and VH:CD ratio (P < 0.001), whereas NC birds showed greater crypt depth (P < 0.001). Similarly, in the cecum, PC birds showed greater villus height (P < 0.001), whereas NC birds exhibited greater crypt depth and the lowest VH:CD ratio (P < 0.001).

***Day 26.*** TBA0.1 promoted greater villus height in the duodenum (P < 0.001), and PC also showed lower crypt depth and a higher VH:CD ratio (P < 0.001), whereas in the jejunum the NC treatment presented the lowest villus height and greatest crypt depth (P < 0.001) and PC the highest VH:CD ratio (P < 0.001). In both the ileum and cecum, birds in the PC treatment showed greater villus height, lower crypt depth, and higher VH:CD ratios than the other treatments (P < 0.001).

***Day 40.*** NC birds showed the lowest villus height in the duodenum (P < 0.001), whereas the supplemented treatments resulted in greater crypt depth (P < 0.001) and PC showed the highest VH:CD ratio compared to TBA0.05 (P = 0.005). In the jejunum, PC and NC birds presented the greatest crypt depth, while NC showed the poorest VH:CD ratio (P < 0.05). In the ileum, only the VH:CD ratio differed among treatments, with the NC treatment showing the lowest value (P < 0.001). In the cecum, TBA0.05 and TBB0.05 resulted in lower villus height and crypt depth (P < 0.001) and higher VH:CD ratios compared to NC treatment (P = 0.020).

### Intestinal pH

The intestinal pH results are presented in Table 6.

**Table 6:** Intestinal segment pH in broiler chickens challenged with dextran sulfate sodium and supplemented with tributyrin.

Age	Segment	Treatments					SEM^1^	p-value
		PC	NC	TBA0.1	TBA0.05	TBB0.05		
**13 d**	**Duodenum**	6.27	6.21	6.22	6.21	6.19	0.011	0.173
	**Jejunum**	6.17 a	6.06 b	6.05 b	6.05 b	6.02 b	0.013	0.005
	**Ileum**	6.68 a	6.21b	6.37ab	6.73 a	6.39 ab	0.054	0.006
	**Cecum**	6.67 a	6.00 b	6.41 ab	6.22 b	6.21 b	0.058	0.001
**21 d**	**Duodenum**	6.16	6.22	6.21	6.19	6.19	0.011	0.519
	**Jejunum**	6.05	6.08	6.09	6.01	6.09	0.020	0.667
	**Ileum**	6.51 b	6.69 ab	6.79 ab	6.76 ab	6.87 a	0.040	0.050
	**Cecum**	6.50	6.66	6.68	6.44	6.40	0.040	0.065
**26 d**	**Duodenum**	6.14	6.14	6.11	6.03	6.12	0.026	0.732
	**Jejunum**	5.91	5.99	5.94	5.88	5.89	0.020	0.433
	**Ileum**	6.76 a	6.57 ab	6.52 ab	6.46 ab	6.23 b	0.050	0.018
	**Cecum**	6.56	6.69	6.58	6.38	6.46	0.043	0.175
**40 d**	**Duodenum**	6.07 a	5.97 a	5.68 b	6.01 a	6.05 a	0.036	0.001
	**Jejunum**	5.87	5.97	5.79	5.88	5.76	0.035	0.311
	**Ileum**	6.84	6.80	6.41	6.79	6.74	0.064	0.262
	**Cecum**	6.46	6.63	6.51	6.38	6.50	0.037	0.313

PC = basal diet without DSS challenge; NC = basal diet with 3.5% DSS challenge; TBA0.1 = 3.5% DSS challenge + basal diet supplemented with 0.1% tributyrin A; TBA0.05 = 3.5% DSS challenge + basal diet supplemented with 0.05% tributyrin A; TBB0.05 = 3.5% DSS challenge + basal diet supplemented with 0.05% tributyrin B.

Data are presented as means of 12 replicate pens per treatment (one bird sampled per pen; n = 12 birds per treatment).

^ab^ Means within a row followed by different superscript letters differ according to Student–Newman–Keuls (SNK) test (P < 0.05).

1 Standard error of the mean.

***Day 13:*** Birds in the PC treatment showed the highest pH in the jejunum (P = 0.005). In the ileum, PC and TBA0.05 also exhibited higher pH values than NC treatment (P = 0.006). In cecum, PC exhibited higher pH values than the NC, TBA0.05 and TBB0.05 treatments (P = 0.001). No differences were observed in the duodenum (P > 0.05).

***Day 21:*** Differences were detected only in the ileum, where birds in the PC treatment showed lower pH values than those in the TBB0.05 treatment (P = 0.050). No differences were observed in the duodenum, jejunum, or cecum (P > 0.05).

***Day 26*.** Only ileal pH differed among treatments, with birds in the PC treatment showing higher pH values than those in the TBB0.05 treatment (P = 0.018). No differences were found in the duodenum, jejunum, or cecum (P > 0.05).

***Day 40.*** Birds receiving the TBA0.1 treatment showed the lowest duodenal pH (P = 0.001). No differences were detected in the jejunum, ileum, or cecum (P > 0.05).

### SCFA concentration in the cecum

The results for SCFA concentration in the cecum are presented below in Table 7.

**Table 7:** Cecal short-chain fatty acid concentrations in broiler chickens at 26 and 40 d of age after dextran sulfate sodium challenge and tributyrin supplementation.

Age	SFCA (mmol/kg)	Treatments					SEM^1^	p-value
		PC	NC	TBA0.1	TBA0.05	TBB0.05		
**26 d**	Acetate	62.54	53.07	63.92	61.00	65.50	3.090	0.765
	Butyrate	11.02	10.45	11.79	13.15	9.09	0.652	0.364
	Proprionate	6.27	8.36	7.20	6.42	7.02	0.361	0.419
**40 d**	Acetate	93.05 a	56.58 b	57.95 b	55.41 b	46.29 b	3.331	<0.001
	Butyrate	13.36	10.71	10.89	12.15	11.51	0.675	0.711
	Proprionate	14.06	10.89	12.06	15.29	10.71	0.573	0.050^2^

PC = basal diet without DSS challenge; NC = basal diet with 3.5% DSS challenge; TBA0.1 = 3.5% DSS challenge + basal diet supplemented with 0.1% tributyrin A; TBA0.05 = 3.5% DSS challenge + basal diet supplemented with 0.05% tributyrin A; TBB0.05 = 3.5% DSS challenge + basal diet supplemented with 0.05% tributyrin B.

Data are presented as means of 12 replicate pens per treatment (one bird sampled per pen; n = 12 birds per treatment) in each age.

^a,b^ Means within a row followed by different superscript letters differ according to Student–Newman–Keuls (SNK) test (P < 0.05).

1 SEM = Standard error of the mean.

2 The reported *p*-value corresponds to an exact value of 0.0503; therefore, no significant differences among treatments were detected.

***Day 26*.** No differences were observed in the concentrations of cecal SCFAs among treatments (P > 0.05).

***Day 40.*** Acetate concentration differed among treatments, with birds in the PC treatment showing higher values than birds in the other treatments (P < 0.001). No differences were observed for the remaining SCFAs (P > 0.05).

## Discussion

The hypothesis of this study was that dietary supplementation with tributyrin would attenuate dextran sulfate sodium-induced intestinal damage and improve intestinal integrity and broiler performance. Taken together, the results confirm this hypothesis, while also indicating that the effects of tributyrin are stage and segment dependent, with a greater impact on the functional recovery of the mucosa after the challenge.

The initial positive effects observed on performance, particularly on body weight at 8 days of age in birds from the highest supplementation treatment and on feed conversion at 13 days of age in birds from all supplemented treatments, compared to birds from the NC treatment, suggest that tributyrin plays a relevant role during the digestive immaturity phase. These initial responses may be explained by the digestive limitations inherent to young birds, which are characterized by reduced pancreatic enzyme secretion and bile salt availability, potentially limiting nutrient digestion and energy utilization (Al-Marzooqi et al., 1999; Ravindran et al., 2016). In this context, tributyrin, which is readily hydrolyzed to release butyrate, may increase butyrate availability in the intestinal environment and potentially contribute to improved intestinal function in young birds (Elnesr et al., 2020; Nari et al., 2020; Lilburn and Loeffler, 2015).

Furthermore, butyrate availability during early rearing period has been associated with to intestinal development, since cecal fermentation, the main source of endogenous butyrate in birds, is still immature during this period (Wu et al., 2018; Pires et al., 2022). These findings are consistent with the previously described biological functions of butyrate, which serves as a preferred energy substrate for enterocytes and has been reported to regulate processes related to epithelial proliferation and differentiation (Salvi and Cowles, 2021; Onrust et al., 2015). Moreover, this period is characterized by intense intestinal growth and high energy demands, providing a biological justification for a stronger response to additives in young birds (Ravindran and Abdollahi, 2021).

After the second challenge cycle, the birds receiving the lower tributyrin A supplementation levels exhibited improved performance, suggesting that tributyrin supplementation may have helped alleviate some of the negative consequences of the intestinal challenge, since the released butyrate undergoes β-oxidation and is converted into acetyl-CoA and used in ATP production (Li et al., 2015). In addition, part of this response can be attributed to the morphometric adaptations of the intestinal epithelium discussed below, especially increased villus height and improvement in the VH:CD ratio, parameters commonly associated with intestinal absorptive capacity (Salvi et al., 2021; Donohoe et al., 2011).

On the other hand, the absence of differences in other performance parameters indicates that the effects of tributyrin are dynamic and phase-dependent (Recharla et al., 2023). With advancing age and physiological maturation of the gastrointestinal tract, including increased digestive enzyme activity and mucosal absorptive efficiency, the magnitude of responses to additives may decrease (Bedford et al., 2022; Sakomura et al., 2004). Furthermore, during the growth phase, functional recovery of digestion may have contributed to partial recovery of digestive efficiency, even if body weight gain was reduced by previous damage. DSS-based enteric challenge models demonstrate inflammation that progresses in a time- and segment-specific manner, and may persist beyond histomorphology, indicating that functional impairment may last or occur without major morphometric differences at a single sampling point (Dal Pont et al., 2021). Furthermore, birds may exhibit reduced feed intake proportional to the reduction in body weight gain, resulting in similar feed conversion but with lower total gain. Enteric challenges are known to reduce apparent energy yield and nutrient absorption, which can suppress body weight gain with varying effects on feed conversion, depending on the balance between intake and digestibility (Kim et al., 2022, 2023).

Histological changes are widely recognized as sensitive markers of mucosal integrity and function (Belote et al., 2019). In the present study, DSS challenge effectively induced histological changes in intestinal segments, indicating the establishment of a damage, as previously reported (Dal Pont et al., 2021; Menconi et al., 2015). In the duodenum, the challenge caused an increase in enterocyte proliferation in the initial phase, a response that has been associated with epithelial adaptation during acute intestinal damage (Sanches et al., 2020). Over time, the supplemented groups exhibit a progressive modulation of this response, with increased goblet cell presence, which may contribute to epithelial protection through mucus production, as previously described (Ismael et al., 2025; Tugnoli et al., 2020). In the jejunum, the effects of the challenge were most evident in the initial phase, with changes consistent with acute damage, followed by a pattern of regeneration in the supplemented groups, evidenced by the recovery of goblet cells and epithelial proliferation, reflecting the intestinal alterations induced by the first challenge cycle (Zhou et al., 2018), potentially influenced by the trophic effects attributed to butyrate supplementation (Haldar et al., 2019). Over time, morphological patterns suggestive of epithelial recovery were observed, contributing to the preservation of intestinal integrity through the proliferation of enterocytes and goblet cells, which is consistent with the established role of butyrate as a preferred energy substrate for intestinal epithelial cells (Gupta et al., 2020; Tugnoli et al., 2020; Allaire et al., 2018).

In distal segments, tributyrin supplementation was associated with responses indicative of preserved mucosal integrity. These effects suggest a sustained action of butyrate in these segments, possibly due to its gradual release along the gastrointestinal tract. Furthermore, the proliferative response of enterocytes in the cecum is consistent with the reported trophic effects of butyrate on intestinal epithelial cells (Tugnoli et al., 2020; Liu et al., 2018).

Another important indicator of intestinal health is epithelial integrity. Intestinal morphometric parameters, including villus height, crypt depth and the VH:CD ratio, are key indicators of absorptive capacity and intestinal function (Marchewka et al., 2021). When analyzing the morphometric results, we observed a deleterious effect of DSS on villus height and a worsening of the VH:CD ratio in all ages and segments evaluated, which may indicate impaired intestinal absorptive capacity. Conversely, tributyrin supplementation was associated with improvements in these parameters, corroborating previous findings (Melaku et al., 2024; Zhao et al., 2022; Liu et al., 2019; González-Ortiz et al., 2019). These improvements reflect enhanced mucosal integrity, which may favor the absorption capacity of nutrients at the affected previously (Hollemans et al., 2020; Elnesr et al., 2020), potentially contributing to improved productive performance.

When we analyzed the VH:CD ratio in isolation, we observed that the effects of tributyrin were more pronounced from 21 days of age onwards. At 13 days, the first cycle of DSS gavage had ended; therefore, birds were likely undergoing an adaptation phase, exhibiting the first responses to the intestinal damage. After ingestion, tributyrin is not completely hydrolyzed, being gradually converted to butyrate in the intestine through the action of pancreatic lipases, resulting in a progressive release along the intestinal tract (Yang et al., 2023). This characteristic of tributyrin allows a greater amount of butyrate to reach the distal portion of the gastrointestinal tract (Wang et al., 2021a). This mechanism may contribute to explain the morphometric results found in our study, especially in the ileum and cecum, where we observed greater improvement of the VH:CD ratio at all ages for the supplemented treatments compared to the CN. Other authors have also reported improvements in the villus:crypt ratio of broilers supplemented with tributyrin at different ages (Gong et al., 2021; Hu et al., 2021; Wang et al., 2021b).

Regarding intestinal pH, treatment effects varied across intestinal segments and sampling ages. Although differences were observed in specific segments, particularly in the ileum, the responses did not follow a consistent pattern throughout the experimental period. These findings suggest that luminal pH was influenced by the combined effects of the DSS challenge and dietary treatments, making it difficult to attribute the observed changes exclusively to tributyrin supplementation. Given that luminal pH is influenced by multiple factors, further studies are needed to clarify the mechanisms underlying these responses.

The absence of changes in cecal SCFA concentrations was expected, as previously described (Pearce et al., 2025). These findings suggest that the butyrate released from tributyrin was utilized along the gastrointestinal tract, sustaining its local trophic and regenerative effects on the intestinal mucosa, as evidenced by the improvements in morphometric and histological parameters. In contrast, the reduction in acetate may reflect impaired cecal fermentation induced by DSS. Previous studies have shown that DSS challenge decreases cecal microbiota diversity and disrupts microbial fermentation in broiler chickens (Zou et al., 2019). Moreover, acetate is the predominant SCFA in the chicken cecum (Jiang et al., 2013); therefore, alterations in microbial fermentative activity may be more readily reflected in acetate concentrations. Although tributyrin has been reported to modulate gut microbiota (Hu et al., 2021; Zou et al., 2019), this mechanism was not evaluated in the present study and cannot be confirmed based on cecal acetate concentrations alone, which remained reduced in all DSS-challenged groups.

Collectively, our results demonstrate that tributyrin supports intestinal integrity under enteric challenge conditions, improving productive performance and intestinal morphology in broiler chickens. The highest level of supplementation demonstrated greater consistency in the recovery of intestinal integrity after the challenge, particularly regarding morphometric improvements observed in the second half of the production phase. Therefore, supplementation with 0.1% may help maintain intestinal integrity and promote long-term recovery in birds subjected to severe challenges, resulting in outcomes similar to those of unaffected birds.

Although the present study demonstrated that dietary tributyrin alleviated DSS-induced intestinal damage through histopathological and morphometric assessments, the lack of molecular analyses should be recognized as a limitation. Therefore, future studies integrating histological, molecular, microbiota profiling, and functional assessments are needed to provide a more comprehensive understanding of the mechanisms underlying the beneficial effects of tributyrin in DSS-challenged broilers.

## Conclusion

These results reinforce the potential of tributyrin as a nutritional strategy to mitigate DSS-induced damage and preserve mucosal morphology under enteric challenge. In particular, supplementation with 0.1% tributyrin proved to be an effective nutritional strategy for improving intestinal morphology and productive performance in broiler chickens.

### Declaration of AI and AI-assisted technologies

During the preparation of this manuscript, the authors used Scispace AI for grammatical review and fluency of the English language. After using this tool/service, the authors reviewed and edited the content as needed and assume full responsibility for the content of the published article.

## Author Contributions

Gabriel R. Braga contributed to the experimental design, conducted the experiment, analyzed and interpreted the results, organized the dataset, and prepared the manuscript; Maria R. S. Farias contributed to the scientific discussion of the results and critically revised the manuscript; Lucimauro Fonseca contributed to the analysis and interpretation of experimental data, evaluated the results, and contributed to manuscript preparation; Mariana A. Nascimento contributed to the experimental procedures, evaluation of experimental results, and interpretation of collected data; Wender V. Fernandes contributed to the experimental procedures, evaluation and interpretation of experimental results; Ana L. C. Barros contributed to the experimental procedures, evaluation and interpretation of experimental results; Fernanda S. Marks contributed to experimental data collection, sample preparation, evaluation and interpretation of collected data, and manuscript revision; Lukas Bauer contributed to the study conception, development of the experimental methodology, interpretation of results, and critical revision of the manuscript; Victor D. Naranjo contributed to the study conception, development of the experimental methodology, interpretation of results, and critical revision of the manuscript and Melissa I. Hannas conceived and supervised the study, obtained funding, contributed to experimental design and methodology development, interpreted the results, prepared and critically revised the manuscript, and coordinated the research project. All authors read and approved the final manuscript.

## Disclosures

The authors declare that they have no competing interest relative with this manuscript.
